# Risk Factors for Poor Prognosis of Spinal Cord Injury without Radiographic Abnormality Associated with Cervical Ossification of the Posterior Longitudinal Ligament

**DOI:** 10.1155/2022/1572341

**Published:** 2022-02-17

**Authors:** Bing Cao, Fengning Li, Yifan Tang, Lianshun Jia, Xiongsheng Chen

**Affiliations:** Spine Center, Department of Orthopaedics, Shanghai Changzheng Hospital, Second Military Medical University, 415 Fengyang Road, Huangpu District, Shanghai, China

## Abstract

**Purpose:**

To investigate the factors associated with the prognosis of spinal cord injury without radiographic abnormality (SCIWORA) accompanied by cervical ossification of the posterior longitudinal ligament (C-OPLL).

**Methods:**

We retrospectively investigated 287 patients with SCIWORA associated with C-OPLL, who were admitted within 30 days after trauma to our facility between August 2014 and August 2018. All patients were divided into the good or poor prognosis group. Patient demographics were analyzed. Besides, occupying ratio on CT and spinal cord high signal changes in MRI T2WI were measured and recorded. Multivariate linear regression was applied to analyze the correlation of prognosis with spinal cord high signal changes in MRI T2WI, cause of injury, and occupying ratio.

**Results:**

Occupying ratio of ossification mass was 43.5 ± 10.7% in the poor prognosis group and 27.3 ± 7.7% in the good prognosis group. The occurrence rate of high signal changes in MRI T2WI was 84.2% in the poor prognosis group and 41.3% in the good prognosis group. Poor prognosis was correlated with high occupying ratio and spinal cord high signal changes in MRI T2WI. In the patient with SCIWORA associated with C-OPLL, ROC curve of occupying ratio showed 30% as a predictor for the poor prognosis. Among the 92 patients with occupying ratio ≤ 30%, poor prognosis was observed in 5 cases (5.4%), whereas in the 72 cases with occupying ratio > 30%, poor prognosis was seen in 33 cases (45.8%). Postoperative AIS grade at final follow-up in occupying ratio > 30% group was significantly worse.

**Conclusions:**

Patients suffering from SCIWORA with C-OPLL have poor prognosis when they have higher occupying ratio of ossification mass and spinal cord high signal changes in MRI T2WI. The cut-off value of occupying ratio for predicting the poor prognosis was 30% in patients with SCIWORA associated with C-OPLL.

## 1. Introduction

Spinal cord injury without radiographic abnormality (SCIWORA) is a syndrome that involves spinal cord injury (SCI) without evidence of spine fracture or dislocation on plain radiographs or computed tomography (CT), characterized by low energy damage and mostly incomplete quadriplegia [1–3]. Besides children, SCIWORA is also common among middle-aged and elderly people [4]. The incidence of SCIWORA is increasing, and the prevalence of cervical ossification of the posterior longitudinal ligament (C-OPLL) among SCIWORA cases is alarmingly high [5].

The prevalence of C-OPLL among SCI patients was higher than the general prevalence rate of C-OPLL. C-OPLL may increase the risk of SCI associated with minor injury. There are many reports on SCI associated with C-OPLL; however, the precise mechanism remains unknown. Kwon et al. [6] reported that high cord compression ratio was related to neurological prognosis in patients with SCI associated with C-OPLL. Previous work on the risk factors of prognosis in patients with SCI associated with C-OPLL was unable to eliminate the impact of different types of spine fracture or dislocation on prognosis. However, to the best of our knowledge, risk factors of prognosis in patients with SCIWORA associated with C-OPLL have been rarely studied.

We retrospectively analyzed patients with SCIWORA associated with C-OPLL. According to the neurological outcomes assessed by the American Spinal Injury Association (ASIA) impairment scale (AIS) grade at admission and discharge, we defined absence of improvement of AIS grade as poor prognosis and improved neural function based on AIS grade as good prognosis [7]. All the patients received anterior or posterior cervical decompression and fusion. We compared the general condition, cause of injury, surgical treatment, occupying ratio, and high signal changes in T2W MRI between the two groups in order to identify risk factors for poor prognosis of SCIWORA associated with C-OPLL after surgical treatment.

## 2. Materials and Methods

### 2.1. Patients

We retrospectively investigated 287 patients with SCIWORA associated with C-OPLL, who were admitted within 30 days after trauma to our facility between August 2014 and August 2018. The inclusion criteria were history of trauma, presence of ossification of posterior longitudinal ligament in cervical spine on cervical CT images, and absence of major fracture or dislocation of the cervical spine on X-rays or CT. Patients with a previous history of cervical SCI, surgery, tumor, or tuberculosis were excluded. Finally, 164 patients (mean age: 56.3 years) who had undergone surgical decompression were included in this study. All patients presented symptoms of cervical spinal cord injury (SCI) and MRI manifestations consistent with symptoms secondary to cervical spinal cord compression. For each patient, SCIWORA was confirmed by clinical syndromes, physical examinations, MRI demonstration of cord compression, and CT-confirmed absence of major fracture or dislocation of the cervical spine. Patients with impaired neurological function and persistent cervical spinal cord compression were indicated for surgery [8].

All patients were divided into the poor prognosis group (absence of improvement based on the AIS grade, 38 cases) and the good prognosis group (improved neural function based on AIS grade, from A to B or C or D or E, from B to C or D or E, from C to D or E, and from D to E, 126 cases) [7]. The two groups were compared in terms of age, gender, comorbidities, cause of injury, the time from injury to operation, preoperative neurologic status, morphology of OPLL, surgical treatment, intraoperative blood loss, occupancy ratio, and spinal cord high signal changes in MRI T2WI (Table 1).

### 2.2. Surgical Treatment

The surgical procedure of anterior cervical ossified posterior longitudinal ligament en bloc resection (ACOE) has been previously reported by us [9]. The surgical procedure of anterior cervical discectomy and fusion (ACDF) has been previously described [10]. The surgical procedure of anterior hybrid fusion surgery has been previously described [11]. The surgical procedure of posterior total laminectomy with fusion (PTLF) has been previously described [12].

### 2.3. Postoperative Treatment

All patients received antibiotics to prevent infection, 30 minutes before the operation and 24 hours after the operation. The drainage tubes were removed based on the drainage volume. Excessive neck rotation, extension, and flexion were avoided. The patients were encouraged to cough and complete functional exercise. After surgery, all patients were immobilized with a Philadelphia collar for 12 weeks.

### 2.4. Clinical Evaluation and Radiological Assessment

**Figure 1:**
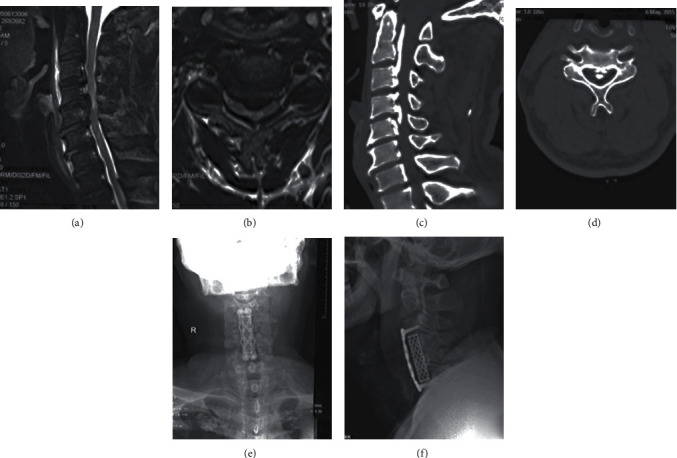
A 54-year-old male patient with SCIWORA associated with C-OPLL received anterior cervical decompression and fusion. (a) Intramedullary high signal intensity (SI) in preoperative sagittal T2-weighted MRI scan. (b) Compressed spinal cord on preoperative axial T2-weighted MRI scan. (c, d) Cervical ossification of the posterior longitudinal ligament on sagittal and axial CT scans. (e, f) Anterior cervical decompression and fusion at C3-6 segment on postoperative plain radiographs (anteroposterior and lateral).

**Figure 2:**
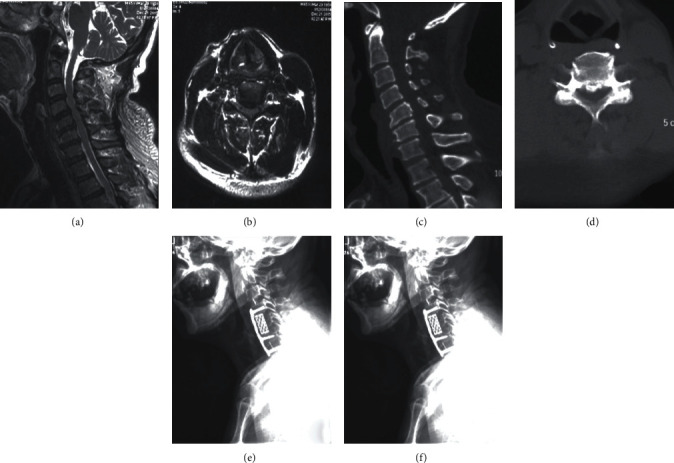
A 65-year-old male patient with SCIWORA associated with C-OPLL received anterior cervical decompression and fusion. (a) No intramedullary high signal intensity (SI) in preoperative sagittal T2-weighted MRI scan. (b) Compressed spinal cord on preoperative axial T2-weighted MRI scan. (c, d) Cervical ossification of the posterior longitudinal ligament on sagittal and axial CT scans. (e, f) anterior cervical decompression and fusion at C4-7 segment on postoperative plain radiographs (anteroposterior and lateral).

Postoperative outcomes at final follow-up were assessed and recorded according to the AIS grade [7]. Follow-up duration ranged from 2 to 3 years. The thickness of OPLL mass and anteroposterior diameter of spinal canal was measured on axial CT image. The occupancy ratio was defined as the biggest ratio of OPLL mass to anteroposterior diameter of spinal canal on the axial CT image [13]. According to the standard of spinal cord high signal changes in MRI T2WI [14], all patients were divided into two groups based on the presence or absence of spinal cord high signal changes in MRI T2WI (Figure 1 and Figure 2).

### 2.5. Statistical Analysis

Statistical analyses were performed using SPSS17.0 statistical software. The data are expressed as the mean ± standard deviation. Differences in continuous variables were compared using the Student's unpaired *t*-test, while differences in categorical variables were compared using the chi-square test. Then, a multiple logistic regression model yielding odds ratios (ORs) and 95% confidence intervals (CI) was used to identify predictors of poor prognosis (nonfunctional improvement based on the AIS grade) on discharge. Receiver operating characteristic (ROC) curves were drawn, and the area under the ROC curve along with its corresponding 95% CI provided a measure of overall validity. The cut-off value of the occupancy ratio for predicting the postoperative poor prognosis was determined using the ROC analysis and Youden's index [15]. A *p* value < 0.05 was considered to be statistically significant. All tests were two-tailed.

## 3. Results

### 3.1. Patient Characteristics

There were no significant differences in age, gender, comorbidities, preoperative neurologic status, morphology of OPLL, and intraoperative blood loss between the two groups (Table 1).

There was no difference in the distribution of cause of injury between the two groups. There was no difference in the distribution of surgical treatment between the two groups. There was significant difference in the distribution of spinal cord high signal changes in MRI T2WI between the two groups according to *χ*^2^ test. The average occupancy ratio of patients in the poor prognosis group was 43.5 ± 10.7%, which was significantly higher than that in the good prognosis group (27.3 ± 7.7%).

### 3.2. Multivariate Analysis

Multivariate analysis was conducted for spinal cord high signal changes in MRI T2WI, cause of injury, and occupancy ratio. According to the stepwise regression method, the results showed that occupancy ratio and spinal cord high signal changes in MRI T2WI were associated with poor prognosis. The final regression model was as follows:

(1) $$\begin{array}{c} \text{Poor prognosis}=1/\left(1+\exp\left[-\left(0.9694+4.679\times \text{High signal of spinal cord}+0.489\times B+0.074\times C+0.023\times D+0.385\times E+0.126\times \text{Occupancy ratio}\right)\right]\right)\\ \left(\text{R}-\text{Square}=0.3874,\mathrm{Max}-\text{rescaled R}-\text{Square}=0.5858\right) \end{array}$$

The poor prognosis after SCIWORA associated with C-OPLL was significantly correlated with high occupancy ratio and spinal cord high signal changes in MRI T2WI (Table 2).

### 3.3. ROC Curve

**Figure 3:**
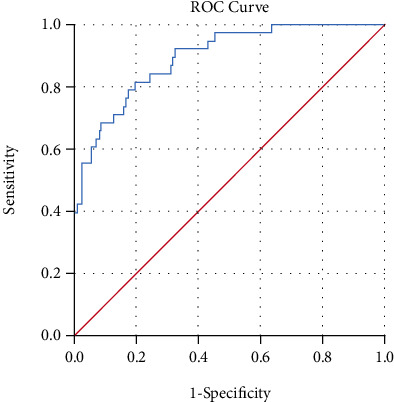
The receiver operating characteristic (ROC) curve of occupancy ratio for predicting the prognosis in all patients. The area under the ROC curve was 0.894.

In the patients with SCIWORA associated with C-OPLL, ROC curve analysis showed occupancy ratio > 30% to be the value to maximize the power of ossification thickness as a predictor for the poor prognosis (*p* < 0.001, AUC = 0.894, 95% CI 0.839 − 0.950, sensitivity = 97%, specificity = 61%) (Figure 3).

164 patients were divided into 2 groups: occupancy ratio > 30% group and occupancy ratio ≤ 30% group. There were 72 patients (43.9%, 72 of 164) in the occupancy ratio > 30% group and 92 patients (56.1%, 92 of 164) in the occupancy ratio ≤ 30% group. There were no significant differences in age, gender, comorbidities, and cause of injury between the two groups (Table 3). The prognosis in the occupancy ratio > 30% group was significantly worse than that in the occupancy ratio ≤ 30% group (*p* < 0.001) (Table 4).

**Figure 4:**
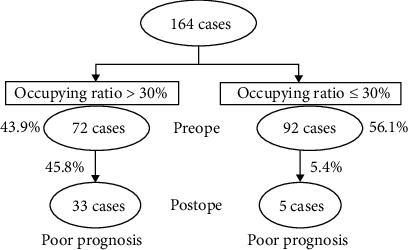
The classification based on the occupancy ratio in the patient with SCIWORA associated with C-OPLL. Postoperative prognosis between occupancy ratio > 30% and occupancy ratio ≤ 30% group.

In 164 patients with SCIWORA associated with C-OPLL, the incidence of poor prognosis is higher in the group with occupancy ratio more than 30% (Figure 4). Among the 72 patients with occupancy ratio more than 30%, poor prognosis was observed in 33 patients (45.8%), whereas in the 92 patients with occupancy ratio less than 30%, poor prognosis was seen in 5 patients (5.4%) (Figure 4).

## 4. Discussion

For patients with SCIWORA accompanied by C-OPLL, the correlation between occupancy ratio and poor prognosis remains unclear. Kwon et al. [6] reported that the degree of spinal cord compression correlated with prognosis of SCI. Jung et al. [16] concluded that severe spinal cord compression was a risk factor for acute progression in patients with SCI accompanied by C-OPLL after minor trauma. However, according to other studies, spinal cord compression did not correlate with poor prognosis of SCI. Okada et al. [17] found no correlation between the occupancy ratio and the severity of paralysis at the time of injury in patients with SCI associated with C-OPLL.

In this study, the poor prognosis group had higher occupancy ratio and higher occurrence of spinal cord high intensity on MRI T2WI than the good prognosis group. The result of multivariate analysis showed that higher occupancy ratio and higher occurrence rate of spinal cord intensity correlated with poor prognosis. For patients with SCIWORA accompanied by C-OPLL, OPLL was the static factor, and the possible mechanisms were as follows: (1) direct injury from the ossification mass. At the time of traumatic injury, the spinal cord under static compression of ossification mass could be abruptly pinched by ossification mass, resulting in secondary damage of spinal cord, which induced neurological deficits and deterioration [6]. (2) The buffering and protective potential of cerebrospinal fluid (CSF) decreased. For C-OPLL patients, especially those with severe spinal canal stenosis, their CSF zone was narrowed. In traumatic injuries, without effective buffering and protection, the traumatic force will be directly conducted to the spinal cord, which may induce a concussion of the spinal cord [6]. Therefore, when patients with C-OPLL had traumatic injury, cervical SCI without effective protective potential of CSF may lead to a poor prognosis.

However, Okada et al. [17] did not find any correlation. We hypothesized that the possible reason for this difference was that the severity of paralysis was affected by the degree of spinal cord compression and the degree of traumatic force [18]. The Okada et al. study [17] only included 13 patients with SCI associated with C-OPLL, who did not make comparison of variables within groups, such as cause of injury and degree of traumatic force. For patients with SCI, especially those with severe cervical spinal canal stenosis, surgical treatment may be necessary [3, 18–21]. For patients with SCIWORA, surgical treatment may achieve better neurological prognosis compared with conservative treatment [22]. A previous study [20] included patients with SCI who had received conservative treatment, which may be the reason why all the patients uniformly achieved poor prognosis. Therefore, our study ensured the preconditions of same type of injury and same degree of traumatic force and surgical treatment and subsequently conducted a follow-up of the patients with SCIWORA accompanied by C-OPLL ranging from 2 to 3 years. Anterior and posterior approaches are the mainstay of treatment for cervical spinal cord compression at present [23]. Chen et al. reported that the absence of cervical lordosis may cause insufficient posterior shifting of the spinal cord after the posterior approach procedure because the spinal cord likely still bowstrings against anterior OPLL [24]. To those massive OPLL patients with canal-occupying ratio > 50% − 60%, the anterior surgery group showed significantly better prognosis than the posterior group, while to those canal-occupying ratio < 50% − 60%, the postoperative JOA score and recovery were similar between the two groups [25]. We recommend the anterior approach for the treatment of OPLL when patients with occupying ratio ≥ 50% or absence of cervical lordosis. Nagamoto et al. also performed anterior selective stabilization combined with laminoplasty for massive OPLL, with a canal occupying ratio ≥ 50% and hill-shaped ossification [26], which may be an alternative treatment. However, posterior approaches can shorten the operation time and reduce intraoperative blood loss [27]. We recommend posterior cervical spine surgery for patients with worse health status. For patients with occupying ratio < 50% and cervical lordosis, spinal surgeons may choose either anterior or posterior approaches.

The cut-off value of occupancy ratio for predicting the poor prognosis was 30% in the patient with SCIWORA associated with C-OPLL. In the current study, we found 30% to be the cut-off ratio for predicting the poor prognosis in the patients with SCIWORA associated with C-OPLL. For C-OPLL patients, especially those with occupancy ratio > 30%, their CSF zone was narrowed severely, which may lead to lack of effective buffering and protection.

The occurrence rate of high signal changes in spinal cord in MRI T2WI was 84.2% in the poor prognosis group and 41.3% in the good prognosis group, with significant difference. High signal changes in the spinal cord in MRI T2WI originate from dynamic damage of traumatic force and static damage of continuous spinal cord compression. Dynamic damage may be induced by of traumatic force, which abruptly pinches the spinal cord by inner structures of the spinal canal, and causes sudden neurological injury. Static damage may originate from mechanical damage of direct compression from ossification of the posterior longitudinal ligament [6]. Kwon et al. [6] found that high signal changes in MRI T2WI were associated with poor prognosis in patients with SCIWORA. Machino et al. [8, 28] found that high signal changes in T2W MRI were associated with preoperative severe paralysis and poor postoperative neurological recovery status. Therefore, based on the existence of high signal changes in T2W MRI, an accurate prediction of the prognosis of patients with SCIWORA associated with C-OPLL cannot be made. However, some patients with high signal spinal cord changes in MRI T2WI and high occupancy ratio had good prognosis. Of the 72 patients with more than 30% occupancy ratio, 31 patients had signal changes and good prognosis. The possible reason was that progressive change is observed in high signal intensity lesions in the intramedullary region. High signal spinal cord changes in MRI T2WI can be observed in patients with early edema and demyelination. Decompression of the spinal cord by surgery can restore blood circulation in the spinal cord and reduce swelling, thus reducing or normalizing the high signal intensity in the intramedullary region. Patients with high signal intensity in the intramedullary region in MRI T2WI can have good prognosis after surgery when they are at early stage of edema and demyelination [29–31]. Ramanauskas et al. [32] reported that in the early stage, the spinal cord demonstrated high signal intensity in MRI T2WI, while in the late stage, low signal intensity in MRI T1WI was noted. Considering the absence of early appearance, we did not take the signal changes in the intramedullary region in MRI T1WI into consideration. There is no correlation between the morphology of OPLL and prognosis in this study. Kim et al. reported that the morphology of OPLL was not associated with a poor prognosis [33].

Consequently, high occupancy ratio and high signal changes in T2W MRI are significant risk factors for developing severe paralysis in patients with SCIWORA associated with C-OPLL. The cut-off value of occupancy ratio for predicting the poor prognosis was 30% in patients with SCIWORA associated with C-OPLL. The present study had several limitations: (1) the number of subjects was relatively small. (2) Multiple factors are associated with the prognosis of C-OPLL. This study only focused on the occupancy ratio and high signal changes in T2W MRI, which may have ignored other possible significant risk factors. Since this study was retrospective and based on a small sample size, large-scale and prospective studies are necessary and warranted.

## 5. Conclusion

This study evaluated the occupancy ratio and high signal changes in T2W MRI in patients with SCIWORA associated with C-OPLL.

The poor prognosis group exhibited a higher occupancy ratio and a higher incidence of high signal changes in T2W MRI than the good prognosis group.

The cut-off value of occupancy ratio for predicting the poor prognosis was 30% in patients with SCIWORA associated with C-OPLL.

## Figures and Tables

**Table 1:** Clinical characteristics and neurological outcomes according to the prognosis.

Variable	Poor prognosis group (*n* = 38)	Good prognosis group (*n* = 126)	*p* value
Age ($\bar{x}\pm s$, years)	55.0 ± 12.0	56.7 ± 11.0	0.551
Gender (male/female, cases)	28/10	94/32	0.909
Comorbidity			
Hypertension	9	32	0.831
DM	6	13	0.356
Smoking	2	8	0.806
Heart disease	4	16	0.720
Peripheral vascular disease	3	9	0.876
Cause of injury			0.778
Ground level falls	20	56	
Bicycle accidents	5	21	
Low-speed MVA	8	26	
High-speed MVA	5	23	
Preoperative neurologic status			<0.001
AIS grade A	7	1	
AIS grade B	10	6	
AIS grade C	10	42	
AIS grade D	11	72	
AIS grade E	0	5	
Morphology of OPLL			0.778
Local	20	56	
Segmental	5	21	
Continuous	8	26	
Mixed	5	23	
Surgical treatment			0.100
ACDF	16	56	
ACOE	7	21	
Anterior hybrid fusion	3	26	
PTLF	12	23	
Intraoperative blood loss $\left(\bar{x}\pm s,\text{ml}\right)$	244.7 ± 92.6	199.1 ± 118.6	0.325
High signal changes in T2W MRI	32/6	52/74	<0.001
Occupancy ratio (%)	43.5 ± 10.7	27.3 ± 7.7	0.004

**Table 2:** Multivariate logistic regression analysis results for the prognosis and other factors.

Prognosis	OR	95% CI	*p* value
High signal changes in T2W MRI			
No	Reference		
Yes	4.679	1.464-14.948	0.009
Preoperative neurologic status			
AIS grade A	Reference		
AIS grade B	0.489	0.037-6.481	0.588
AIS grade C	0.074	0.007-0.768	0.029
AIS grade D	0.023	0.002-0.272	0.003
AIS grade E	0.385	0.027-5.465	0.481
Occupancy ratio	0.126	0.039-0.410	<0.001

**Table 3:** Comparison of demographic characteristics in the patients according to the classification based on the occupancy ratio.

Variable	Occupancy ratio > 30% (*n* = 72)	Occupancy ratio ≤ 30% (*n* = 92)	*p* value
Age ($\bar{x}\pm s$, years)	57.1 ± 11.0	55.7 ± 11.4	0.578
Gender (male/female, cases)	53/19	69/23	0.840
Complication			
Hypertension	16	25	0.467
DM	6	16	0.091
Cause of injury			0.884
Ground level falls	32	44	
Bicycle accidents	11	15	
Low-speed MVA	17	17	
High-speed MVA	12	16	

**Table 4:** Comparison of postoperative prognosis in the patient with SCIWORA associated with C-OPLL.

Variable	Occupancy ratio > 30% (*n* = 72)	Occupancy ratio ≤ 30% (*n* = 92)	*p* value
Time from injury to operation ($\bar{x}\pm s$, days)	16.8 ± 7.4	16.1 ± 8.5	0.137
Surgical treatment			0.105
ACDF	37	53	
ACOE	12	19	
Anterior hybrid fusion	4	9	
PTLF	19	11	
Intraoperative blood loss $\left(\bar{x}\pm s,\text{ml}\right)$	225.0 ± 106.4	197.7 ± 119.6	0.900
Preoperative neurologic status			<0.001
AIS grade A	8	0	
AIS grade B	9	6	
AIS grade C	10	29	
AIS grade D	10	53	
AIS grade E	36	4	
Postoperative neurologic status at final follow-up			<0.001
ASI grade A	7	0	
AIS grade B	9	2	
AIS grade C	10	6	
AIS grade D	10	15	
AIS grade E	36	69	
Poor prognosis	33	5	<0.001

## Data Availability

If there is any need of the underlying data, I would upload the underlying data as soon as possible.

## Additional Points

*Device Status/Drug Statement*. The manuscript submitted does not contain information about medical device(s)/drug(s).
